# Correlates of Measured Prehypertension and Hypertension in Latina Women Living Along the US–Mexico Border, 2007–2009

**DOI:** 10.5888/pcd11.140233

**Published:** 2014-10-23

**Authors:** Hala Madanat, Marisa Molina, Hena Din, Rachel Mintle, Elva M. Arredondo, John P. Elder, Kevin Patrick, Hector Lemus, Veronica Medina, Guadalupe X. Ayala

**Affiliations:** Author Affiliations: Marisa Molina, San Diego State University Research Foundation, San Diego Prevention Research Center, San Diego, California; Hena Din, San Diego Prevention Research Center and the Institute for Behavioral and Community Health of the San Diego State University Research Foundation, San Diego, California; Rachel Mintle, Hector Lemus, Graduate School of Public Health, San Diego State University and San Diego Prevention Research Center and the Institute for Behavioral and Community Health of the San Diego State University Research Foundation, San Diego, California; Elva M. Arredondo, Guadalupe X. Ayala, Graduate School of Public Health, San Diego State University and San Diego Prevention Research Center and the Institute for Behavioral and Community Health of the San Diego State University Research Foundation, San Diego, California; John P. Elder, San Diego Prevention Research Center and the Institute for Behavioral and Community Health of the San Diego State University Research Foundation, San Diego, California, and Department of Family and Preventive Medicine, University of California, San Diego, California; Kevin Patrick, Department of Family and Preventive Medicine, University of California, San Diego, California; Veronica Medina, San Ysidro School District, San Diego, California.

## Abstract

**Introduction:**

Although Latinos have lower hypertension rates than non-Latino whites and African Americans, they have a higher prevalence of undiagnosed and uncontrolled hypertension. Research on predictors of hypertension has mostly focused on intrapersonal factors with no studies assessing the combined influence of intrapersonal, interpersonal, and environmental factors. The purpose of this study was to assess a broad range of correlates including intrapersonal, interpersonal, and environmental factors on measured blood pressure category (nonhypertensive, prehypertensive, and hypertensive) in a sample of Latina women residing in San Diego, California.

**Methods:**

This cross-sectional study used baseline data from the San Diego Prevention Research Center’s *Familias Sanas y Activas* program, a *promotora*-led physical activity intervention. The sample was 331 Latinas who self-selected into this program. Backward conditional logistic regression analysis was conducted to determine the strongest correlates of measured blood pressure category.

**Results:**

Logistic regression analysis suggested that the strongest correlates of prehypertension were soda consumption (odds ratio [OR] = 1.34, [1.00–1.80], *P* ≤ .05) and age (OR = 1.03, [1.00–1.05], *P* ≤ .05). The strongest correlates of hypertension were soda consumption (OR = 1.92, [1.20–3.07], *P* ≤ .01), age (OR = 1.09, [1.05–1.13], *P* ≤ .001), and measured body mass index (OR = 1.13, [1.05–1.22], *P* ≤ .001). All analyses controlled for age and education. No interpersonal or environmental correlates were significantly associated with blood pressure category.

**Conclusion:**

Future research should aim to further understand the role of soda consumption on risk for hypertension in this population. Furthermore, interventions aimed at preventing hypertension may want to focus on intrapersonal level factors.

## Introduction

More than a quarter of deaths among US Latinas are attributable to diseases in which hypertension is a risk factor. In 2010, 20.9% of deaths among Latinas were due to cardiovascular disease, 6.0% to stroke, and 2.4% to kidney disease (1). Hypertension, combined with other highly prevalent risk factors such as low levels of physical activity, sedentary lifestyle, and obesity, increases risk of death among Latinas (2).

Most research studies examining correlates of hypertension have largely focused on intrapersonal factors such as diet (3), physical activity (4), and acculturation (5). Some have assessed interpersonal factors and neighborhood characteristics. For example, studies on interpersonal factors have found that emotional support provided by friends, family, or partners or attending group support sessions decreased a person’s risk for hypertension (6). Studies that focused on neighborhood characteristics have found that neighborhood safety, cohesion, and walkability, and availability of healthy food in a neighborhood were associated with lower blood pressure among residents (7). For Latinos, living in neighborhoods with higher concentrations of immigrants and Latinos also decreased the risk of hypertension (8). However, to our knowledge, no study has examined the combined influence of intrapersonal, interpersonal, and environmental factors on risk of hypertension among Latinas.

The purpose of this study was to examine the effects of a broad range of factors associated with prehypertension and hypertension in a group of Latinas who self-selected to participate in a free physical activity program. This study is framed within the socioecological framework, which is widely used in public health. The socioecological framework is often conceptualized as 5 levels of influence: intrapersonal, interpersonal, organizational, environmental, and policy. In this study, factors representing intrapersonal, interpersonal, and environmental levels of influence on hypertension were examined.

## Methods

### Study design and data source

This cross-sectional study used baseline data from the San Diego Prevention Research Center’s *Familias Sanas y Activas* program, a *promotora*-led physical activity intervention. The South Bay region of San Diego County has a large proportion of Latinos (63.8%), of which 32.0% are Latina. In 2011, the median household income was $43,903, and the median age of Latinas was 30.6 years. Approximately 38% of the Latina population graduated from high school (9).

In the spring of 2007, bilingual research assistants recruited South Bay community members to participate in the study. The research assistants used the Physical Activity Readiness Questionnaire (PAR-Q) (10) to screen potential participants for eligibility; those who responded yes to any of the PAR-Q questions were given a Physical Activity Readiness medical examination form (PARmed-X) and instructed to obtain medical clearance before undergoing baseline measurements and participating in program activities.

A total of 531 people were screened for eligibility. Of these, 517 gave written informed consent and 387 completed the baseline assessment. Men (n = 16) were excluded because of the small sample size. Women previously diagnosed with hypertension (n = 37) were excluded because of unknown medication use, as were women for whom a previous diagnosis of hypertension was unknown (n = 2). One woman did not complete the blood pressure evaluation. The final analytic sample was 331 Latinas aged 18 to 72 years. Further details regarding recruitment and training are described elsewhere (11). The institutional review boards of San Diego State University and the University of California at San Diego approved the study.

### Measures

Seated resting blood pressure was taken with an Omron automatic blood pressure monitor according to the National Health and Nutrition Examination Survey Anthropometry Procedures Manual (12). Participants were asked to sit without talking for 5 minutes with feet flat on the ground. All jewelry or watches were removed before the test was administered. The measurement was taken on a participant’s bare left arm using the appropriate size cuff, with the arm placed at the level of the heart. A reading was taken and recorded for each participant. Blood pressure was categorized on the basis of standard clinical cut points (systolic/diastolic): normal (<120/80 mm Hg), prehypertensive (120–139/80–89 mm Hg), and hypertensive (≥140/≥90 mm Hg) (13).

Height was measured and recorded in centimeters with a stadiometer, and weight was measured and recorded in kilograms with a digital scale. BMI was calculated by dividing weight in kilograms by height in meters squared, by using the mean of 3 nonconsecutive measurements. Time of day and clothing worn were recorded (14). BMI was categorized into normal (BMI <25.0), overweight (BMI 25.0–29.9) and obese (BMI >29.9).

Physical activity was assessed using the Global Physical Activity Questionnaire. This scale has been validated with adult Latinos (15). Fifteen questions inquired about both moderate- and vigorous-intensity physical activity in 3 domains: work, traveling from place to place (transportation), and recreational activities (leisure-time). For this study, only moderate-to-vigorous leisure-time physical activity was used. The number of minutes of moderate-to-vigorous leisure-time physical activity was dichotomized into “meets or does not meet physical activity recommendations” (≥150 minutes of moderate physical activity or ≥75 minutes of vigorous physical activity per week in leisure time activity).

Consumption of fast foods was assessed with a question from the 2007 Behavioral Risk Factor Surveillance System (BRFSS) survey (16). The question asked how many times in a typical week fast food was eaten for breakfast, lunch, or dinner. Fast food included food from fast food restaurants, lunch wagons, and vending machines. The number of times fast food was eaten in a typical week was used as a continuous variable.

Smoking behavior was assessed by using an item from the 2005 BRFSS survey (17). It assessed frequency of current smoking with the following response options: “every day,” “some days,” and “not at all.” Responses were dichotomized to “nonsmoker” and “smoker,” which collapsed “every day” and “some days.”

The fruit and vegetable consumption questions were adapted from the 2005 BRFSS survey (17). Two questions asked about the number of servings of fruits and vegetables the participant consumed in a typical day with responses ranging from 1 serving to 5 or more servings. Serving sizes for both fruits and vegetables were described to the participant. Servings of fruits and vegetables were combined and retained as a continuous variable.

The question about soda consumption was adapted from the 2005 BRFSS survey (17). Participants were asked how many 12-oz cans of regular soda they consumed in a typical day, not including diet soda. Number of sodas was used as a continuous variable for analysis.

Participants were asked how many hours of television (TV) they watched on a typical workday. This question was taken from the 2005 BRFSS survey (17). The variable “hours of TV watched” was used as a continuous variable for analysis.

Social support for engaging in physical activity was assessed by using questions selected from a 13-item scale developed by Sallis et al (18). The selected questions examined instrumental and emotional support received from friends, family, and partner. Friend and family social support was assessed by 3 questions each; a sample question was “During the past month, how often did your friends (family) offer to do physical activity with you?” Five options were provided from “never” to “very often.” Mean scores were obtained, and a higher score demonstrated greater perceived social support from friends (α = .87) and family (α = .80).

Partner support was evaluated using 5 modified items from the Sallis scale (18). Participants were asked how frequently they received partner support: “During the past month, how often did your partner take over chores so you had time to be physically active?” The 4 response options were “never,” “rarely,” “sometimes,” or “often.” Mean scores were obtained; a higher score indicated greater perceived partner support for physical activity (α = .85).

Neighborhood cohesion was evaluated with 4 statements adapted from the Neighborhood Cohesion Scale (19). The scale measures attraction to neighborhood, degree of neighboring, and psychological sense of community. Participants were asked to rate their agreement to statements such as “The relationships I have with my neighbors mean a lot to me.” Response options included 1, very true; 2, sort of true; or 3, not at all true. The scale was reverse-coded for analysis, so a higher mean score represented greater perceived neighborhood cohesion.

Neighborhood safety was measured with 9 statements examining the following: perceptions about neighborhood traffic and speed, lighting, visibility, pedestrian safety, crime, and stray dogs. Participants were asked to rate their agreement with statements such as “There is so much traffic along streets in my neighborhood that it makes it difficult or unpleasant to walk.” Response options ranged from 1 (strongly disagree) to 5 (strongly agree). A mean score was calculated, with a higher score indicating greater perceived neighborhood safety.

Demographic information (ie, age, marital status, employment, education, and place of birth) was obtained by using questions from the 2005 BRFSS survey and from previous studies (17). Marital status was dichotomized into married or living as married versus other. Employment was dichotomized to employed for wages or self-employed versus all others (retired, homemaker, student, out of work, or unable to work). Education was categorized as high school educated or greater versus less than a high school education. Place of birth was categorized as US-born versus other. Income was assessed with the question, “What is your household’s monthly income from all sources?” Participants could either fill in an exact amount or choose from a monthly range (1, less than $500; 2, $500 to $999; 3, $1,000 to $1,499, and so on). The 2010 federal poverty guidelines, determined by family size and annual income, were used as a cut-off. The annual income variable was dichotomized to above or below the federal poverty guidelines. The acculturation scale was adapted from the Short Acculturation Scale for Hispanics (20). The modified scale, using 8 of the original 12 questions, examines language use and media use. Each language and media-use question had 5 response options (1, only Spanish; 2, more Spanish than English; 3, both equally; 4, more English than Spanish; or 5, only English). A mean score was calculated such that a higher score indicated greater English language use (α = .88).

### Statistical analyses

Analyses were conducted using SPSS Statistics version 19 (IBM Corp). Analyses of variance (ANOVA) and Pearson χ^2^ tests were conducted to identify significant differences by blood pressure category on interpersonal, intrapersonal, and environmental factors; post hoc analyses examined individual group differences (*P* < .05). Because of the exploratory nature of these analyses, backward conditional logistic regression analysis was conducted to determine the strongest correlates of blood pressure category. Only correlates identified as significant in the post hoc analyses were included in the logistic regression model. Age and education were included in the regression model as control variables.

## Results

Participants’ blood pressure categories are presented by sample demographic characteristics (Table 1). A total of 331 women with a mean age of 39.9 years (standard deviation [SD], 10.6 y) participated in baseline measures. Participants whose blood pressure was considered hypertensive were significantly older than those in the prehypertensive and normal groups (*P* < .001). Most participants were foreign-born (77.3%) and married (70.9%); 56.5% had received a high school education or greater. The percentage of women who completed high school was significantly higher in the nonhypertensive group than in the others (*P* = .008). Forty-eight percent of participants reported an annual household income above the calculated federal poverty guidelines of $24,000.

**Table 1:** Overall Demographic Characteristics of Participants by Blood Pressure Category, South San Diego County, 2007–2009

Demographic Variable	Total Sample (N = 331)	Normal (N = 130)	Prehypertensive (N = 148)	Hypertensive (N = 53)	*P* Value
Age in years, mean (SD)	39.9 (10.6)	37.2 (10.3)	40.2 (10.3)	45.7 (9.9)	<.001
Married, % (n)	70.9 (234)	71.5 (93)	67.3 (48)	79.2 (42)	.257
Employed, % (n)	40.7 (134)	36.2 (47)	44.5 (65)	41.5 (22)	.366
High school education or more, % (n)	56.5 (186)	66.9 (87)	50.7 (74)	47.2 (25)	.008
Income above federal poverty guidelines, % (n)	48.1 (137)	52.3 (58)	46.4 (58)	42.9 (21)	.484
Foreign-born, % (n)	77.3 (255)	75.5 (98)	76.9 (113)	83.0 (44)	.529
Acculturation score, mean (SD)	1.96 (0.9)	1.92 (0.8)	2.01 (0.9)	1.94 (0.9)	.651

Abbreviation: SD, standard deviation.

Of the health behaviors selected, significant differences by blood pressure category were observed for soda consumption only (Table 2). Soda consumption was significantly lower in the nonhypertensive than in the prehypertensive group (*P* < .024), and the hypertensive group (*P* < .041).

**Table 2:** Differences in Intrapersonal, Interpersonal, and Perceived Environmental Factors by Blood Pressure Category, South San Diego County, 2007–2009

Hypertension Risk Factors	Total Sample (N = 331)	Normal (N = 130)	Prehypertensive (N = 148)		Hypertensive (N = 53)	*P* Value
	% (SD)					
**Intrapersonal factors**						
**Health measures**						
BMI, kg/m^2^, mean (SD)	30.1 (6.2)	27.8 (5.1)		31.3 (6.6)^a^	32.0 (6.2)^b^	<.001
**Health behaviors**						
Met recommended leisure-time MVPA guidelines, % (n)	45.9 (152)	48.5 (63)	43.2 (64)		47.2 (25)	.670
No. of times had fast food in last week, mean (SD)	1.48 (1.4)	1.65 (1.3)	1.34 (1.4)		1.45 (1.3)	.250
Smoker, % (n)	12.1 (4)	10.0 (13)	13.6 (20)		13.2 (7)	.634
No. of servings of fruits and vegetables consumed daily, mean (SD)	4.9 (2.2)	4.7 (2.3)	5.1 (2.2)		5.0 (2.3)	.353
No. of sodas consumed daily, mean (SD)	0.7 (1.1)	0.5 (0.7)	0.8 (1.3)^a^		0.8 (0.9)^b^	.036
Hours of television on a typical work day, mean (SD)	2.9 (3.4)	3.1 (4.3)	2.8 (2.9)		2.3 (1.5)	.343
**Interpersonal factors: social support, mean (SD)**						
Family support	2.8 (1.2)	2.8 (1.1)	2.8 (1.1)		2.6 (1.2)	.549
Friend support	2.6 (1.2)	2.4 (1.2)	2.7 (1.2)		2.6 (1.2)	.263
Partner support	2.6 (0.9)	2.5 (0.9)	2.6 (0.9)		2.6 (1.0)	.627
**Perceived environmental factors: neighborhood characteristics, mean (SD)**						
Neighborhood cohesion	2.2 (0.6)	2.1 (0.6)	2.3 (0.5)^a^		2.4 (0.5)^b^	.006
Neighborhood safety	2.8 (0.5)	2.9 (0.6)	2.8 (0.5)		2.8 (0.5)	.316

Abbreviations: SD, standard deviation; BMI, body mass index; MVPA, moderate to vigorous physical activity.

a Prehypertensive group is significantly different from the normal group at the *P* < .05 level of significance.

b Hypertensive group is significantly different from the normal group at the *P* < .05 level of significance.

The mean BMI for participants was 30.1 (SD = 6.2), which is considered obese (14). BMI was significantly lower in the nonhypertensive group than in the prehypertensive (*P* < .001) and hypertensive groups (*P* < .001). Significant differences between blood pressure categories were not evident for meeting physical activity recommendations, smoking, fruit and vegetable consumption, or hours of television watched.

Mean social support scores were as follows: friend support, 2.57 (SD = 1.20, range 1–5); family support, 2.79 (SD = 1.16, range 1–5); and partner support, 2.59 (SD = 0.90, range 1–4). We found no significant differences in social support between blood pressure categories. Neighborhood cohesion, with a higher score representing more cohesion, had a mean score of 2.22 (SD = 0.55, range 1–3). Neighborhood cohesion was significantly lower in the nonhypertensive group than in the prehypertensive (*P* = .02) and hypertensive groups (*P* = .004). Neighborhood safety had a mean score of 2.82 (SD = 0.52, range 1–4), with no differences emerging from the various blood pressure categories.

Logistic regression analysis suggested that significant correlates of hypertension were intrapersonal factors including soda consumption (OR = 1.92; 95% CI, 1.20–3.07), BMI (OR = 1.13, 95% CI, 1.05–1.22), and age (OR = 1.09; 95% CI, 1.05–1.13) (Table 3). Similarly, for prehypertension, the strongest correlate was soda consumption (OR = 1.34; 95% CI, 1.00–1.80). Older age and having a higher BMI score were also significantly associated with prehypertension (OR = 1.03; 95% CI, 1.00–1.05 and OR = 1.11; 95% CI, 1.05–1.17, respectively). High school education was inversely associated with prehypertension but was not significant (OR = 0.63; 95% CI, 0.37–1.07). For the environmental correlates, greater neighborhood cohesion was associated with being prehypertensive, but the association was not significant.

**Table 3:** Multivariate Analysis of Intrapersonal, Interpersonal, and Environmental Correlates of Hypertension and Prehypertension, South San Diego County, 2007*–*2009

Hypertension status	OR (95% CI)	*P* Value
**Hypertensive**		
**Intrapersonal correlate**		
Age	1.09 (1.05–1.13)	<.001
Soda consumed on a typical day	1.92 (1.20–3.07)	.006
High body mass index (≥25.0 kg.m^2^)	1.13 (1.05–1.22)	.001
**Prehypertensive**		
**Intrapersonal correlates**		
Older age	1.03 (1.00–1.05)	.044
High school education or more	0.63 (0.37–1.07)	.089
Soda consumed on a typical day	1.34 (1.00–1.80)	.047
High body mass index (≥25.0 kg.m^2^)	1.11 (1.05–1.17)	<.001
**Environmental correlate**		
Greater neighborhood cohesion	1.58 (0.97–2.58)	.065

Abbreviations: OR, odds ratio, CI, confidence interval.

## Discussion

At the intrapersonal level, age, BMI, and soda consumption were positively associated with measured prehypertension and hypertension. Consistent with findings from other studies, older age increased the risk of both prehypertension and hypertension (21). Additionally, having a higher BMI increased the odds of having both prehypertension and hypertension, which is consistent with previous research demonstrating a positive association between BMI and blood pressure (22). For example, previous research shows that hypertension is 2.5 to 3 times higher in obese Latinos than in average-weight Latinos. In addition, although education was not significantly associated with blood pressure category for prehypertension, the trend indicated a negative association, as identified in previous research (23).

Greater consumption of soda was associated with both prehypertension and hypertension. This finding is consistent with previous research suggesting that soda consumption plays a role in increasing blood pressure and risk for other cardiometabolic risk factors (24). Although diet soda consumption was not measured in this study, Cohen et al found that both regular soda and diet soda were associated with increased risk of hypertension (25). Research suggests that decreasing consumption of sugar-sweetened beverages can reduce blood pressure (26). This is especially significant because approximately one-fourth of adults in the United States consume at least 1 sugar-sweetened beverage per day (27). Policy makers are attempting to reduce consumption by ensuring access to potable water and increasing taxes on sugar-sweetened beverages (28).

Finally, research suggests that physical activity can lower blood pressure and is considered a modifiable lifestyle factor for hypertension and other cardiovascular diseases (13). The lack of significant association in this study may be due to our use of self-reported physical activity. Although a validated measurement tool was used, time spent participating in leisure-time physical activity may have been inaccurately reported, a common occurrence with self-reported physical activity (7).

At the interpersonal level, social support was not a significant correlate of hypertension or prehypertension. Previous studies have shown inverse associations between cardiovascular disease risk factors and social support: those who reported more social support had a lower prevalence of cardiovascular disease risk factors including elevated systolic blood pressure (29). Particularly in the Latino community, social support has shown a positive effect on some health behaviors (29). Our findings may indicate that support for physical activity is not as important as other behaviors, such as healthy eating.

At the environmental level, neighborhood cohesion was significantly associated with prehypertension, but not hypertension. Specifically, an increased sense of cohesion was associated with a higher blood pressure category. The direction of this association is surprising and contradictory to previous studies. One previous study found an inverse association between neighborhood characteristics and hypertension (7). Neighborhoods with higher social cohesion and greater walkability had a lower probability of having residents with hypertension. Few studies have explored the connection between specific neighborhood characteristics such as cohesion and hypertension. Although findings of our study do not support previous findings, they provide evidence for continuing to explore the influence of individuals’ perceived environment on their health. In this study, the result may reflect that the desire for a more cohesive environment may actually increase blood pressure in this population.

Several limitations should be noted. Because ours was a cross-sectional study, causal inferences cannot be made. Generalizability of results is limited to adult Latina women who self-selected into a program to improve their health and who consequently may be more health-conscious. These women are also not representative of the general Latina population in the United States because of the geographic location in which they were recruited. As previously mentioned, this study used self-reported physical activity, which overestimates actual physical activity. Using devices such as accelerometers to gather accurate data on physical activity may alter the results (15,30). The PAR-Q requires that a medical release form be obtained for participants who have been diagnosed with high blood pressure. This requirement may have decreased the number of people with high blood pressure in the study sample if they did not or could not obtain a medical release form. Finally, because we used BRFSS questions to measure soda consumption, diet soda consumption was not assessed. Because previous research identified both regular and diet soda as predictive of hypertension (25), future research should assess this relationship in this population.

Despite these limitations, this study is one of the first to our knowledge to examine intrapersonal, interpersonal, and environmental factors associated with hypertension in a US–Mexico border Latino community. Furthermore, the study provides additional support for research linking soda consumption and health outcomes. Results of this study indicate that modifying individual-level factors remains important for prevention of hypertension. Research should also continue to investigate the influence of neighborhood characteristics on blood pressure, as this research is limited in the Latino community.

## References

[1] Heron M . Deaths: leading causes for 2010. Natl Vital Stat Rep 2013;62(6):1–96. 24364902

[2] Zambrana RE , Ayala C , Pokras OC , Minaya J , Mensah GA . Disparities in hypertension-related mortality among selected Hispanic subgroups and non-Hispanic white women ages 45 years and older — United States, 1995–1996 and 2001–2002. Ethn Dis 2007;17(3):434–40. 17985494

[3] Wang L , Manson JE , Gaziano JM , Buring JE , Sesso HD . Fruit and vegetable intake and the risk of hypertension in middle-aged and older women. Am J Hypertens 2012;25(2):180–9. 10.1038/ajh.2011.186 21993367 PMC3258456

[4] Whelton SP , Chin A , Xin X , He J . Effect of aerobic exercise on blood pressure: a meta-analysis of randomized, controlled trials. Ann Intern Med 2002;136(7):493–503. 10.7326/0003-4819-136-7-200204020-00006 11926784

[5] Teppala S , Shankar A , Ducatman A . The association between acculturation and hypertension in a multiethnic sample of US adults. J Am Soc Hypertens 2010;4(5):236–43. 10.1016/j.jash.2010.07.001 20728423

[6] Schulz U , Pischke CR , Weidner G , Daubenmier J , Elliot-Eller M , Scherwitz L , Social support group attendance is related to blood pressure, health behaviors, and quality of life in the Multicenter Lifestyle Demonstration Project. Psychol Health Med 2008;13(4):423–37. 10.1080/13548500701660442 18825581

[7] Mujahid MS , Diez Roux AV , Morenoff JD , Raghunathan TE , Cooper RS , Ni H , Neighborhood characteristics and hypertension. Epidemiology 2008;19(4):590–8. 10.1097/EDE.0b013e3181772cb2 18480733

[8] Viruell-Fuentes EA , Ponce NA , Alegría M . Neighborhood context and hypertension outcomes among Latinos in Chicago. J Immigr Minor Health 2012;14(6):959–67. 10.1007/s10903-012-9608-4 22527740

[9] American Community Survey. Population and housing narrative profile 2008–2012 American Community Survey 5-Year Estimates. http://www.census.gov/acs/www/data_documentation/2012_narrative_profiles/. Accessed May 14, 2014.

[10] Canadian Society for Exercise Physiology. PAR-Q forms. 2002. http://www.csep.ca/english/view.asp?x=698. Accessed May 5, 2014.

[11] Ayala GX ; San Diego Prevention Research Center Team. Effects of a promotor-based intervention to promote physical activity: Familias Sanas y Activas. Am J Public Health 2011;101(12):2261–8. 10.2105/AJPH.2011.300273 22021294 PMC3222415

[12] National Health and Nutrition Examination Anthropometry Procedures Manual. Atlanta (GA): US Department of Health and Human Services, Centers for Disease Control and Prevention; 2002. http://www.cdc.gov/nchs/data/nhanes/nhanes_07_08/manual_an.pdf. Accessed May 14, 2014.

[13] High blood pressure fact sheet. Atlanta (GA): US Department of Health and Human Services, Centers for Disease Control and Prevention; 2011;1–4. http://www.cdc.gov/dhdsp/data_statistics/fact_sheets/fs_bloodpressure.htm. Accessed May 14, 2014.

[14] Flegal KM , Carroll MD , Ogden CL , Curtin LR . Prevalence and trends in obesity among US adults, 1999–2008. JAMA 2010;303(3):235–41. 10.1001/jama.2009.2014 20071471

[15] Hoos T , Espinoza N , Marshall S , Arredondo EM . Validity of the Global Physical Activity Questionnaire (GPAQ) in adult Latinas. J Phys Act Health 2012;9(5):698–705. 22733873 10.1123/jpah.9.5.698 PMC3743722

[16] Centers for Disease Control and Prevention. BRFSS 2007 survey data and documentation. Atlanta (GA): US Department of Health and Human Services, Centers for Disease Control and Prevention; 2007. http://www.cdc.gov/brfss/annual_data/annual_2007.htm. Accessed May 14, 2014.

[17] Centers for Disease Control and Prevention. BRFSS 2005 survey data and documentation. Atlanta (GA): US Department of Health and Human Services, Centers for Disease Control and Prevention; 2005. http://www.cdc.gov/brfss/annual_data/annual_2005.htm. Accessed May 14, 2014.

[18] Sallis JF , Grossman RM , Pinski RB , Patterson TL , Nader PR . The development of scales to measure social support for diet and exercise behaviors. Prev Med 1987;16(6):825–36. 10.1016/0091-7435(87)90022-3 3432232

[19] Seidman E , Allen L , Aber JL , Mitchell C . Development and validation of adolescent-perceived microsystem scales: social support, daily hassles, and involvement. Am J Community Psychol 1995;23(3):355–88. 10.1007/BF02506949 7572836

[20] Marin G , Sabogal F , Marin BV , Otero-Sabogal R , Perez-Stable EJ . Development of a short acculturation scale for Hispanics. Hisp J Behav Sci 1987;9(2):183–205. 10.1177/07399863870092005

[21] Yoon SS , Burt V , Louis T , Carroll MD . Hypertension among adults in the United States, 2009–2010. NCHS Data Brief 2012;(107):1–8. 23102115

[22] Hubert HB , Snider J , Winkleby MA . Health status, health behaviors, and acculturation factors associated with overweight and obesity in Latinos from a community and agricultural labor camp survey. Prev Med 2005;40(6):642–51. 10.1016/j.ypmed.2004.09.001 15850860

[23] Loucks EB , Abrahamowicz M , Xiao Y , Lynch JW . Associations of education with 30 year life course blood pressure trajectories: Framingham Offspring Study. BMC Public Health 2011;11(1):139. 10.1186/1471-2458-11-139 21356045 PMC3053249

[24] Dhingra R , Sullivan L , Jacques PF , Wang TJ , Fox CS , Meigs JB , Soft drink consumption and risk of developing cardiometabolic risk factors and the metabolic syndrome in middle-aged adults in the community. Circulation 2007;116(5):480–8. 10.1161/CIRCULATIONAHA.107.689935 17646581

[25] Cohen L , Curhan G , Forman J . Association of sweetened beverage intake with incident hypertension. J Gen Intern Med 2012;27(9):1127–34. 10.1007/s11606-012-2069-6 22539069 PMC3515007

[26] Chen L , Caballero B , Mitchell DC , Loria C , Lin PH , Champagne CM , Reducing consumption of sugar-sweetened beverages is associated with reduced blood pressure: a prospective study among United States adults. Circulation 2010;121(22):2398–406. 10.1161/CIRCULATIONAHA.109.911164 20497980 PMC2892032

[27] Park S , Pan L , Sherry B , Blanck HM . Consumption of sugar-sweetened beverages among US adults in 6 states: Behavioral Risk Factor Surveillance System, 2011. Prev Chronic Dis 2014;11:E65. 10.5888/pcd11.130304 24762529 PMC4008944

[28] The CDC guide to strategies for reducing the consumption of sugar-sweetened beverages. Atlanta (GA): US Department of Health and Human Services, Centers for Disease Control and Prevention; 2010. http://www.cdph.ca.gov/SiteCollectionDocuments/StratstoReduce_Sugar_Sweetened_Bevs.pdf. Accessed May 14, 2014.

[29] Shelton RC , McNeill LH , Puleo E , Wolin KY , Emmons KM , Bennett GG . The association between social factors and physical activity among low-income adults living in public housing. Am J Public Health 2011;101(11):2102–10. 10.2105/AJPH.2010.196030 21330588 PMC3193546

[30] Blanchard CM , McGannon KR , Spence JC , Rhodes RE , Nehl E , Baker F , Social ecological correlates of physical activity in normal weight, overweight, and obese individuals. Int J Obes (Lond) 2005;29(6):720–6. 10.1038/sj.ijo.0802927 15795751

